# Marine Biodiversity Conservation Planning in the Indo-Pacific Convergence Zone Based on Ecological Spatial Analysis

**DOI:** 10.3390/biology14060700

**Published:** 2025-06-14

**Authors:** Linlin Zhao, Tingting Li, Bailin Cong, Bei Wang, Kaiyu Liu, Shenghao Liu

**Affiliations:** 1 Marine Ecology Research Center, First Institute of Oceanography, Ministry of Natural Resources, Qingdao 266061, China; zhaolinlin@fio.org.cn (L.Z.); litingting@fio.org.cn (T.L.); biolin@fio.org.cn (B.C.); wangbei@fio.org.cn (B.W.); 2 School of Advanced Manufacturing, Fuzhou University, Jinjiang 362251, China; liukaiyu@fio.org.cn

**Keywords:** biodiversity hotspots, marine protected areas, habitats, anthropogenic activities, conservation gaps, spatial analysis

## Abstract

Marine biodiversity is of critical importance to global ecosystems. The Indo-Pacific Convergence Zone (IPCZ), a global marine biodiversity hotspot, faces escalating threats from human activities and climate change. This underscores the pressing need to develop effective conservation strategies for marine biodiversity in the IPCZ. This study integrates spatial analysis of ecological sensitivity (coral reefs, mangroves, and seagrass) and anthropogenic pressures (shipping/fishing intensity) to identify biodiversity hotspots and conservation gaps. Using datasets from UNEP-WCMC, OBIS, and Global Fishing Watch, we applied GIS-based multi-criteria evaluation to 5408 grid cells (0.5° resolution) across the IPCZ. Results revealed that 14.7% of the study area constitutes biodiversity hotspots, primarily in coastal Philippines, Indonesia’s Lesser Sunda Islands, and northern Australia. However, only 6% of the IPCZ is currently protected, with merely 13.88% of hotspots overlapping existing marine protected areas (MPAs). Anthropogenic pressure hotspots (e.g., Malacca Strait) showed limited spatial overlap with biodiversity hotspots, suggesting species displacement from high-disturbance zones. Priority conservation areas were delineated by balancing ecological significance and economic activity conflicts. We propose targeted strategies, including buffer zones, seasonal no-take areas, and green shipping technologies, to reconcile conservation with sustainable development. This framework provides actionable insights for enhancing MPA networks in biogeographic transition zones.

## Simple Summary

Marine life in the Indo-Pacific Convergence Zone—a vital global biodiversity hotspot—is under growing threat from human activity and climate change. To protect these fragile ecosystems, we mapped areas of high ecological importance alongside regions facing intense human pressures. We found that only 6% of the Indo-Pacific Convergence Zone is currently protected, leaving critical habitats vulnerable. Key biodiversity hotspots, such as coastal areas in the Philippines and Indonesia, showed little overlap with existing marine protected areas. Surprisingly, human activity hotspots were often separate from biodiversity-rich zones, suggesting species may have retreated from heavily disturbed areas. Based on these findings, we propose urgent conservation actions—such as creating buffer zones, seasonal fishing bans, and eco-friendly shipping practices—to safeguard marine life while supporting local economies. This research helps guide global efforts to protect marine biodiversity and ensure healthy oceans for future generations.

## 1. Introduction

Marine biodiversity is a vital component of the global ecosystem, which maintains the balance of marine ecology and provides abundant resources and services for human beings, including food, medicines, and climate regulation [1]. It is estimated that approximately 80% of global species inhabit the oceans, and the health of marine ecosystems directly affects the stability and sustainability of the planet’s ecology [2]. However, marine biodiversity is currently being lost at an unprecedented rate due to the combined pressures of global climate change and human activities. Human impacts, particularly overfishing, habitat destruction, and climate change, are accelerating this loss [3,4]. As of February 2024, the International Union for Conservation of Nature (IUCN) Red List reports that over 46,300 species worldwide are threatened with extinction, many of which are found in marine ecosystems. In response, many countries have implemented measures to mitigate the damage caused by human activities and address the declining state of marine biodiversity.

The Indo-Pacific Convergence Zone (IPCZ) lies at the intersection of the tropical western Pacific Ocean and the eastern Indian Ocean. The region’s complex geology and diverse ocean currents facilitate marine life dispersal, enhancing biodiversity concentration by creating favorable conditions for species to thrive [5]. The IPCZ is recognized as one of the most biodiverse marine regions globally, with its ecosystems and marine species playing a critical role in global marine biodiversity conservation [6,7,8]. The zone spans the exclusive economic zones (EEZs) of Indonesia, the Philippines, Malaysia, Papua New Guinea, the Solomon Islands, and Timor-Leste. This area is known for its rich and diverse ecosystems, including coral reefs, seagrass beds, and mangroves. Notably, the IPCZ hosts a high level of indigenous species diversity, with many species showing some of the highest richness and diversity on Earth [9,10]. It is estimated that the Indo-Pacific Convergence Zone contains approximately 76% of the world’s shallow-water coral species and 37% of the world’s reef fish species [11]. However, the marine biodiversity of the region is increasingly threatened by human activities. Overfishing, coastal development, pollution, and the impacts of climate change, including ocean acidification and temperature rise, have severely affected its ecosystems [12,13]. Overfishing, particularly in tropical areas, has led to the depletion of resources for numerous species [14,15]. Additionally, the risk of collisions between cetaceans and other large marine organisms with ships has increased due to intense shipping activities, posing a significant threat to endangered species [16,17]. These ongoing threats highlight the urgent need for more effective conservation efforts to protect this critical region’s biodiversity.

Marine protected areas (MPAs) are a key strategy for addressing challenges related to marine conservation [18,19]. Establishing MPAs and imposing restrictions on human activities can provide secure habitats for marine organisms, contributing to ecological restoration and the sustainable use of biological resources [20]. In 2010, the Convention on Biological Diversity (CBD) set the Aichi Targets, with Goal 11 aiming to protect 10% of coastal and marine areas by 2020. In 2022, the 15th Conference of the Parties to the Convention on Biological Diversity adopted the Kunming Montreal Global Biodiversity Framework, which further proposes that at least 30% of the world’s marine areas should be included in protected areas by 2030 (the “30 × 30” target). Despite these initiatives, the number and coverage of MPAs in the IPCZ remains insufficient, with poorly distributed and inadequately managed protected areas [21,22]. Current conservation efforts have been ineffective in mitigating the impacts of human activities, highlighting the urgent need for scientifically planned protected area networks to enhance biodiversity conservation in the region [23].

This study aims to propose a systematic framework for marine biodiversity conservation planning within the Indo-Pacific Convergence Zone (IPCZ). The approach integrates ecological criteria and analytical techniques to guide spatial planning. By identifying biodiversity hotspot areas across the Indo-Pacific region using various ecological metrics, the study subsequently conducts spatial overlay analysis with regions experiencing high human activity pressures, such as shipping lanes, fishing hotspots, and marine protected areas. This analysis facilitates the identification of potential conflict zones between economic activities and biodiversity conservation, while also highlighting gaps in marine protection within these overlapping areas. Through a balance between ecological significance and the conflicts arising from economic activities, priority conservation areas are delineated. This multi-criteria approach offers both theoretical insights and practical guidance for marine conservation in the IPCZ, contributing to the advancement of global biodiversity conservation objectives.

## 2. Materials and Methods

### 2.1. Study Area

**Figure 1:**
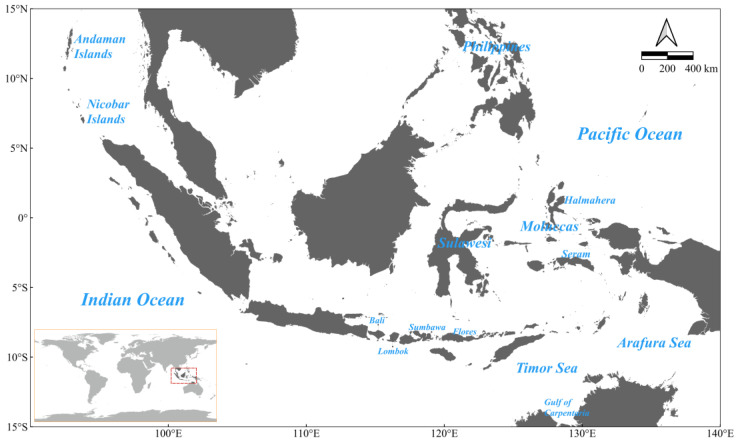
The study area.

The investigated region spans a strategically significant biogeographic transition zone in the Indo-Pacific region (Figure 1). The boundary coordinates of the study area extend from 90° E to 140° E and 15° S to 15° N. This spatial domain encompasses the Indonesian Archipelago, adjacent maritime territories of Southeast Asian nations, and transitional waters between the Indian Ocean and the Western Pacific Ocean. This region was selected to provide comprehensive coverage of key ecological areas in the Indo-Pacific convergence zone, and to provide a basic framework for subsequent biodiversity conservation studies.

### 2.2. Data Collection and Processing

Four key ecological and biological criteria were selected in accordance with the biodiversity conservation area guidelines proposed by Asaad et al. [24]. These criteria include (1) the identification of vulnerable and sensitive habitats, (2) areas with high species richness, (3) the presence of protected species, and (4) the distribution of restricted-range species. The selected criteria, supported by widely available and accessible data, provide a reliable framework for assessing biodiversity conservation, ecosystem health, and species distribution patterns, ensuring a comprehensive analysis of ecological integrity and conservation priorities.

#### 2.2.1. Vulnerable and Sensitive Habitats

Vulnerable and sensitive habitats are generally defined as habitats that are relatively susceptible to natural or anthropogenic threats. Protecting these areas may help reduce disturbance from human activities and increase resilience to natural events. The evaluation of vulnerable and sensitive habitats was conducted using distribution data for the following three critical biological habitats: coral reefs, mangroves, and seagrass beds. These data were compiled by the United Nations Environment Programme World Conservation Monitoring Centre (UNEP-WCMC) in collaboration with a number of international organizations through a combination of technical means (e.g., field surveys, remote sensing techniques, satellite imagery, and habitat modeling). These methods provide strong support for accurately assessing the distribution of vulnerable and sensitive habitats. Examples include habitat maps of coral reefs [25], mangroves [26], and seagrass [27]. These habitats are of significant ecological importance and are particularly vulnerable and sensitive, making them key priorities in biodiversity conservation efforts.

#### 2.2.2. Species Richness

Species richness is an important indicator of biodiversity that reflects the diversity of species within a region. To assess species richness, the following two methods were employed: species occurrence records and species range data. Species occurrence records were obtained from the Ocean Biogeographic Information System (OBIS; Table S1), which provided point-based data on species occurrences within the study area. Species range data were derived from AquaMaps (ver. 10/2019) (Table S2), a tool that generates modeled geographic species distributions. AquaMaps produces probabilistic predictions of species ranges at a resolution of 0.5°, with each raster cell representing the relative suitability for a given species which is indicated by a probability value ranging from 0 to 1. For the purposes of this study, we considered raster cells with occurrence probability values exceeding 0.5 as representing suitable areas for the species’ distribution.

#### 2.2.3. Presence of Protected Species

Protected species (e.g., endangered and vulnerable species) are key indicators of the conservation value of a region’s biodiversity. Not only are these species at high risk of extinction, but their survival is a direct reflection of the health of the region’s ecosystems. To assess the presence of protected species within the designated study area, distribution records were downloaded from the Ocean Biogeographic Information System (OBIS). These records were sourced from the International Union for Conservation of Nature (IUCN) Red List of Threatened Species and the Convention on International Trade in Endangered Species of Wild Fauna and Flora (CITES). To verify the actual presence of these species in the Indo-Pacific convergence region, the OBIS dataset was cross-referenced with additional databases, including FishBase and Sea Life Base, as well as relevant peer-reviewed literature. After rigorous screening and validation, a total of 946 species were identified for further analysis (Table S3). Additionally, the current global conservation status of all identified species was retrieved from the IUCN Red List and the Species + database. This approach ensured the inclusion of species with documented conservation status and geographical relevance, enhancing the accuracy and comprehensiveness of the assessment.

#### 2.2.4. Restricted-Range Reef Fishes

Restricted-range reef fishes tend to have high levels of endemism and vulnerability, and their presence indicates that the area is critical for their survival. Conservation of these species contributes to the maintenance of biodiversity in the region. To assess criteria for restricted-range reef fishes, we extracted distribution records for 373 reef fish species endemic to the Indo-Pacific convergence zone from the Indo-Pacific Reef Fish dataset.

#### 2.2.5. Anthropogenic Pressures

Anthropogenic pressures in marine environments often manifest as an overlap between shipping activities and fisheries capture efforts. To identify these areas of high anthropogenic pressure the Getis-Ord GI* statistical method was employed, which is a robust technique for detecting spatial clusters of high shipping density and intense fishing activity, often referred to as “human pressure hotspots”. The Global Fisheries Watch database was utilized for the purpose of this study, with fishing effort data and shipping data both being drawn from this database. The spatial distribution of these two types of human-induced marine pressure across the Indo-Pacific convergence zone was assessed by calculating mean annual fishing effort (in hours per year) and mean annual vessel stock (in hours per year) for the period 2021–2023. To ensure consistency across the study, all data were standardized to a resolution of 0.5° latitude by longitude. This standardization allowed for uniform spatial analysis, enabling the identification of regions exhibiting high concentrations of both shipping and fishing activities, thereby highlighting areas with significant human-induced marine pressures. This methodological approach provides a clearer understanding of the spatial dynamics of anthropogenic impacts on marine ecosystems in the region.

#### 2.2.6. Marine Protected Areas Distribution Data

To obtain information on the distribution of marine protected areas (MPAs) within the Indo-Pacific Convergence Zone, this study utilized data from the UNEP-WCMC and the IUCN for the year 2023. In addition, the World Database on Protected Areas (WDPA) was consulted to supplement the available information on MPAs. All data were checked in R, with NA values and non-marine data removed to ensure data accuracy and consistency. All datasets, including those pertaining to habitats, species range point records, species ranges, fishing efforts, shipping activities, and MPA locations (as summarized in Table 1), were cross-referenced with the metadata system developed by UNEP-WCMC. For consistency in spatial referencing, all geographic data were standardized to the World Geodetic System 1984 (WGS84) coordinate system, ensuring compatibility across various data sources and facilitating accurate geospatial analysis. This approach provided a comprehensive view of the spatial distribution of MPAs within the study region, enabling a detailed assessment of their coverage in relation to anthropogenic pressures such as fishing and shipping activities.

### 2.3. Spatial Analysis Techniques

To identify key areas with significant biodiversity, spatial analyses were conducted by overlaying multiple data layers representing various environmental variables. All datasets were processed and analyzed using QGIS version 3.30.3 software. A grid-based method, utilizing a half-degree raster resolution, was employed to crop the datasets to the boundaries of the study area. The marine area within the study area was segmented into 5408 raster grids, each covering an area of 55 km × 55 km, which facilitated a systematic and spatially consistent analysis of biodiversity hotspots. Additionally, the overlay of different data layers, including habitat types, species distributions, and anthropogenic pressures, allowed for the identification of regions where biodiversity is potentially under threat while also highlighting areas that may be of high ecological value for conservation planning.

#### 2.3.1. Coverage of Vulnerable and Sensitive Habitats

In assessing the coverage of vulnerable and sensitive habitats, each raster grid cell was categorized into three distinct classes based on the species richness it contained. The classification was as follows: (1) cells containing a single species, (2) cells containing two species, and (3) cells containing three or more species. This classification approach provides a systematic method to quantify and visually represent the spatial distribution of habitat diversity within the study area.

#### 2.3.2. Species Richness and Distribution of Protected Species

The species richness of the area was determined based on both species occurrence records and species ranges derived from modeled geographic distributions. For the species occurrence records, the ntile function in the dplyr package (2.3.2) [28] was used to calculate the number of species in each raster and to classify them into five classes based on their number of species using the quantile method. This method can effectively reflect the concentration trend and discrete degree of species distribution. For species distribution ranges, richness was based on the predicted number of species in each raster. In the context of the study area, the number of species per 0.5° raster varied between 55 and 6642. Consequently, the quantile method was employed to classify the number of species within each raster. Furthermore, an assessment was conducted on the distribution of species of conservation concern, utilizing species occurrence records and analyses analogous to species range richness to determine the number of species, employing the quantile cut method.

#### 2.3.3. Distribution of Restricted-Range Reef Fish

The distribution of restricted-range reef fish was assigned to 0.5° grid cells, and each cell was assessed based on the total number of restricted-range reef fish within it. The values within the grid cells ranged from 0 to 101 species, and the grid cells were classified into five equally spaced classes, namely, class 1 (1–20 species), class 2 (21–40 species), class 3 (41–60 species), class 4 (61–80 species), and class 5 (81–101 species). This classification provides a clear picture of the distribution of reef fish in different regions.

#### 2.3.4. Comprehensive Datasets and Hotspot Analyses

In this study, we utilized ArcGIS 10.7 to generate a comprehensive dataset by overlaying each criteria dataset, with consistent weights assigned to all criteria. Through the analysis of the biodiversity and anthropogenic pressures in each raster, we identified the biodiversity hotspots in the Indo-Pacific convergence zone along with the focus areas of anthropogenic marine pressures. The spatial clustering of phenomena was assessed using the hotspot analysis tool of ArcGIS 10.7, and three categories of hotspots (99%, 95%, and 90% confidence level) were classified. This in turn resulted in the spatial distribution of the hotspots of biodiversity and hotspots of anthropogenic ocean pressures in the Indo-Pacific convergence zone.

#### 2.3.5. Marine Conservation Coverage Assessment

The assessment of current marine protection coverage in the IPCZ was achieved by estimating the proportion of established protected areas within the zone, and by estimating the percentage of overlap between current protected areas and the priority protected areas that had been identified. The spatial distribution patterns of biodiversity hotspots and priority areas for anthropogenic marine pressure were then used to propose areas for the delineation of priority protected areas, with the aim of providing a scientific basis for future marine conservation planning.

## 3. Results

### 3.1. Biodiversity Hotspots Identification

**Figure 2:**
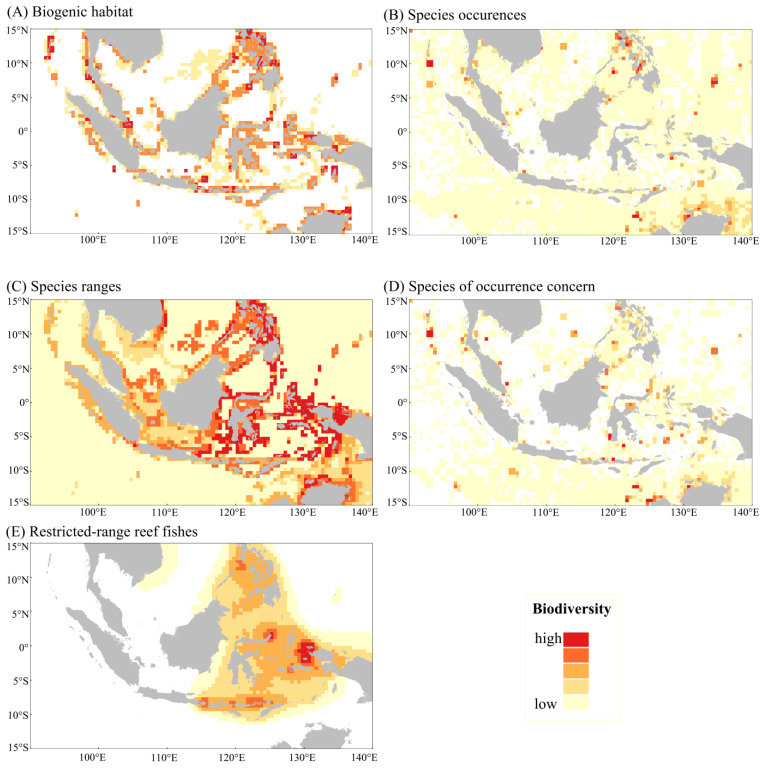
Biodiversity critical areas based on each ecological criterion in the 0.5° cell: (**A**) coral reef, mangrove, and seagrass coverage, (**B**) species richness (number of occurrences) based on 15,045 species, (**C**) species richness based on overlapping ranges of 7231 species, (**D**) species richness based on 946 species of conservation concern, and (**E**) critical areas based on the 373 distribution of endemic reef fishes to delineate areas of significance. White cells = no data.

This study employed a range of ecological criteria to identify and assess biodiversity hotspots within the Indo-Pacific Convergence Zone (Figure 2). To evaluate the vulnerability and sensitivity of key habitats, we utilized join distribution data for the following three critical ecosystems: seagrass meadows, coral reefs, and mangroves (Figure 2A). In the Indo-Pacific Convergence Zone, the three critical habitats of seagrass meadows, coral reefs, and mangrove forests were covered by 17%, 15%, and 7% of hotspots, respectively. Collectively, these habitats encompassed more than 18% of the study area. Specifically, more than 51% of the 0.5° rasters were occupied by a single habitat, 40% of the rasters were covered by two habitats, and only 8% of the rasters were covered by all three habitats simultaneously. It is noteworthy that the southern region of Luzon, the northern expanse of Mindanao, the southernmost point of Peninsular Malaysia, the western region of Java, the northern area of Sulawesi, the Moluccas, and the Raja Ampat Archipelago in Papua, Indonesia, were the only regions where all three habitats were present (Figure 2A).

Species richness was assessed using both distribution records for 15,045 species and model-predicted geographic ranges for 7231 species (Figure 2B,C). The maximum number of species documented within a single grid cell was 1964, while the maximum number of occurrences recorded was 66,396. Of these grid cells, 775 (14%) contained only one species, and 614 (11%) had only one occurrence recorded. The application of the quantile method to the analysis of species occurrence records indicated that a mere 16 grid cells (3%) attained a rank of five, and these regions were identified as exhibiting the highest species richness within the study area. These regions of high species richness were found to be concentrated in the Philippines (Southern Luzon, Mindoro, Cebu, Bohol, and Sulu Archipelago), Malaysia (Semporna Peninsula—Sabah), Indonesia (Northern and Southern Sulawesi, Eastern Bali, Moluccas, and Raja Ampat Islands), Northern Australia, and the Southern Andaman Islands (Figure 2B).

The phyla Radiolaria, Platyhelminthes, and Mollusca exhibited a distribution that encompassed more than 90% of the study area, whereas the corallimorphs constituted a mere 66% of the area. Scleractinians exhibited the highest number of species in a single grid cell (3824), followed by mollusks (2063) (Table 2). An analysis of the overlap in the geographic ranges of predicted species showed that more than 61% of the study area was predicted to be inhabited by fewer than 355 species, and these grid cells were categorized as areas of low species richness. Conversely, a comparatively smaller percentage of grid cells (6%) were predicted to be inhabited by more than 3252 species, and thus these grids were classified as areas of highest species richness. These regions of high species richness are predominantly located along the coastlines of the Philippines (Northern Luzon, Sullivan Sea, Bohol, Mindanao, Palawan, and Sulu Archipelago), Malaysia (Northeastern Sabah), and Indonesia (Northern and Southeastern Sulawesi, Banda Sea, Mollusca, and Papua Raja Ampat Islands) (Figure 2C).

The distribution of 946 species of conservation concern was analyzed to assess criteria related to protected species (Figure 2D). Of these, corallivorous species were the most numerous (710 species), followed by spoke-finned fishes (99 species), planktivorous subclasses (82 species), mammals (36 species), and mollusks (19 species) (Table 3). According to the IUCN Red List criteria, species classified as Near Threatened (186 species), Vulnerable (237 species), Endangered (48 species), and Critically Endangered (29 species) are of conservation concern. A significant proportion of the critically endangered species, specifically 86%, are classified under the subclass Platyhelminthes, as detailed in Table 3 and its accompanying table. According to CITES, 17 species of mammals, 6 species of planktivorous subclasses, and 1 species of bryozoan fish are listed in Appendix I belongs to the CITES, and 838 species are listed in Appendix II belongs to the CITES (Table S2). Notably, all coral orders, particularly hard reef coral species, are included in Appendix II belongs to the CITES of CITES.

The maximum number of species that were found to be protected within a single grid cell was 316, with a maximum occurrence record of 3665. Of these, 1072 grid cells (20 percent) contained only one species. Furthermore, it was determined that nearly 22% (1212 grid cells) of the study area contained at least one species of conservation concern. Most of the area (1975 grid cells, constituting 37%) was classified as an area of low species richness. Conversely, areas comprising less than 1% of the total area exhibited the highest indices and were identified as regions with the highest protected species richness. These areas of high species richness included the Spearmount Islands in Indonesia, the northern Great Barrier Reef in Australia, the southern Andaman Islands, and the northern Marshall Islands (Figure 2D).

The distributions of 373 reef fish species were examined to assess criteria for range-limiting species. A total of 20% of the Coral Triangle is inhabited by coral fishes with restricted distributions. Of these areas, 7% are classified as low endemic species richness areas, and the total number of coral fish with restricted ranges in these areas ranges from 1 to 20 species. A comparatively smaller portion of the study area was classified as high endemic species richness areas, which are inhabited by more than 60 species of coral fishes with restricted distribution ranges. These high richness areas are primarily located in the southern part of Mindoro Island in the Philippines, the northern part of Sulawesi Island, the Four Kings Archipelago, Seram Island, Bali, and the coastal areas of the Nusa Tenggara Islands in Indonesia. The most widely distributed coral fishes, comprising more than 80 species, were observed in the following two locations: the northern part of Sulawesi Island and the Four Kings Islands (Figure 2E).

**Figure 3:**
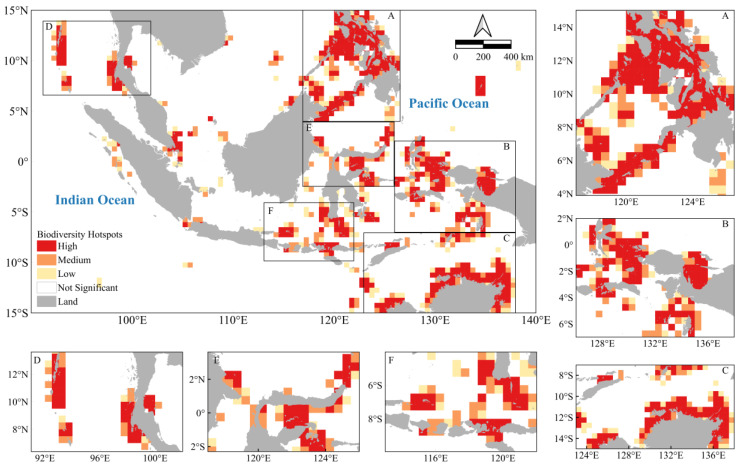
Map of clusters of biodiversity-important cells in the study area. Analyzed by the Hotspot Analysis Tool, this map shows spatially contiguous cells with high biodiversity scores. The analysis clustered the biodiversity scores of neighboring cells into three hotspot categories: high, medium, and low. (**A**): Philippines; (**B**): Moluccas, Halmahera and Seram; (**C**): Arafura Sea and Gulf of Carpentaria; (**D**): Andaman Islands; (**E**): Sulawesi; (**F**): Bali, Lombok and Sumbawa Flores.

By overlaying these different criteria, we identified biodiversity hotspots in the Indo-Pacific convergence zone (Figure 3). It was found that nearly 14.7% of the study area in the Indo-Pacific convergence zone was clustered into biodiversity hotspot areas. Of these, 8.4% were high hotspot areas, 3.8% were medium hotspot areas, and 2.5% were low hotspot areas. These hotspots are widely distributed along the coast of the Philippines (Figure 3A), Andaman and Nicobar Islands, the southern part of Thailand (Figure 3D), Hamahela, Polar Bird Peninsula, Seram, Southeast Islands, Indonesia (Figure 3B), Tomini Bay, Sangaihe Islands, Sulawesi (Figure 3E), Lesser Sunda Islands (Bali, Lombok, Sumbawa, and Flores) (Figure 3E), northern Australia and Timor (Figure 3F), Australia and Timor (Figure 3E), and the northern coast of Australia bordering the Timor Sea, Arafura Sea, and the Gulf of Carpentaria (Figure 3C).

### 3.2. Spatial Distribution of Anthropogenic Marine Activity Pressure

**Figure 4:**
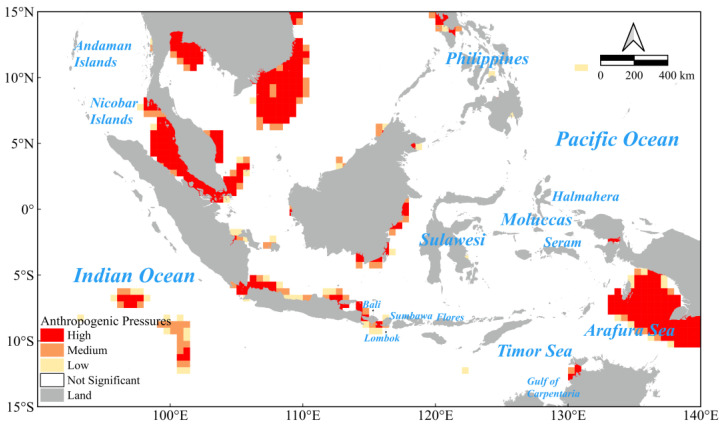
Spatial distribution of anthropogenic pressures on marine activities in the study area.

This study comprehensively assessed the anthropogenic marine activity pressure in the IPCZ and revealed the characteristics of the spatial distribution of disaggregated marine activity pressure in the region through the quantitative analysis of fishing effort and shipping density (Figure 4 and Figure S1). The spatial aggregation analysis of shipping activities demonstrated that the Strait of Malacca, a pivotal conduit between the Indian and Pacific Oceans, exhibits an exceptionally high density of shipping, thereby establishing a pronounced shipping hotspot. Furthermore, the waters surrounding Sumatra and Java, in addition to the northern coastline of Australia, particularly the ports of Darwin and Cairns, also demonstrate high shipping densities (Figure S1A). Conversely, the distribution of fishing effort reveals elevated vessel densities along the coasts of the Philippines (including southern Luzon, Mindoro, Cebu, and around Bohol), northern and south-eastern Sulawesi, around Bali and the Lesser Sunda Islands in Indonesia (including Lombok and Flores), along the southern coast of Thailand (especially along the Andaman Sea), and along the northern coasts of Australia (especially the Timor Sea, the Arafura Sea, and the Carpentaria Sea). The Arafura Sea and the Gulf of Carpentaria represent significant fishing hotspots (Figure S1B).

The integration of vessel shipping density and fisheries fishing effort data has enabled the identification of priority areas of anthropogenic marine pressure in the Indo-Pacific convergence zone (Figure 4). These areas are concentrated along the coast of the Philippines, northern Sulawesi, around Bali, along the southern coast of Thailand, and along the northern coast of Australia. It should be noted that the Strait of Malacca and its surrounding areas exhibit extremely high levels of anthropogenic marine stress due to the dual pressures of both fishing and shipping activities. The identification of these priority areas is imperative for the formulation of targeted marine conservation strategies, which are vital for the effective mitigation of the deleterious impacts of human activities on marine ecosystems.

### 3.3. MPA Coverage and Priority MPA Delineation

**Figure 5:**
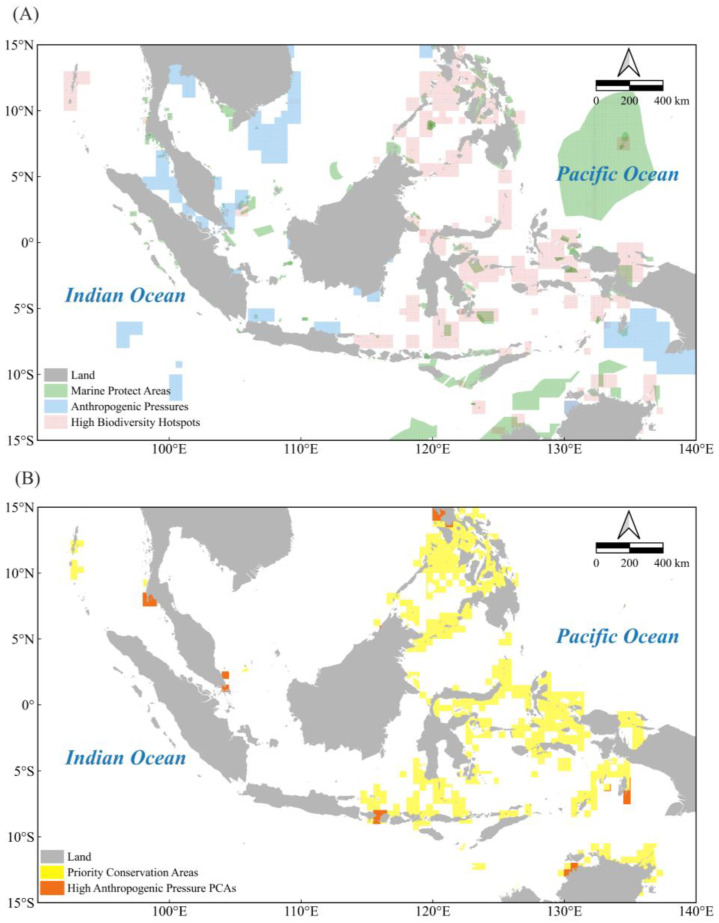
Marine protected area coverage and priority conservation area delineation: (**A**) marine protected areas (green), biodiversity hotspots (red), and distribution of human pressures (blue), and (**B**) priority conservation areas (yellow) and priority conservation areas with high anthropogenic pressures (orange).

This study combined the spatial distribution of biodiversity hotspots and anthropogenic marine activity pressures with the spatial overlay analysis method, based on the 2023 MPA data, to assess MPA coverage in the IPCZ and delineate priority MPAs (Figure 5). The results show that the coverage of existing MPAs in the IPCZ is limited, accounting for only 6% of the study area, and is mainly distributed in the coastal areas of the Philippines, Indonesia, Thailand, and Australia (Figure 5A).

In delineating priority protected areas this study took a comprehensive view of biodiversity conservation, economic factors, and the addition of existing protected areas, with the aim of achieving a harmonious symbiosis between ecological conservation and socio-economic development. The analysis successfully identified priority protected areas in the Indo-Pacific convergence zone (Figure 5B). The yellow areas in the figure are biodiversity hotspots covering the southern Philippines, northern Sulawesi, Bali, Indonesian Hammarhela, the Aurora Peninsula, and the northern coast of Australia. The orange areas are also designated as priority areas for conservation because they are ecologically important despite high anthropogenic pressures. These include the central coast of the Philippines, central and south-west Sulawesi, south-east Indonesia and parts of northern Australia. The selection of these areas is intended to balance the need to conserve biodiversity with human activities through strategic conservation actions, and to contribute to the goals of ecological conservation and sustainable development.

## 4. Discussion

### 4.1. Applying Multiple Criteria to Analyze Biodiversity Hotspots

A significant challenge in implementing biodiversity conservation is the harmonization of the criteria for identifying critical areas and establishing representative biodiversity conservation networks [24,29]. In order to effectively select protected areas, many international environmental initiatives, such as the Convention on Biological Diversity (CBD) and the Global Biodiversity Strategy (GBS), have proposed multiple criteria including biological, ecological, economic, social, and governance criteria to help identify and prioritize areas of significance for conservation [30,31].

However, in recent years, researchers have come to realize that a single ecological criterion may not be able to comprehensively cover all key biodiversity hotspots, especially in the face of complex diversity conservation needs [32]. Consequently, an increasing number of studies have adopted a multi-criteria analysis (MCA) approach, which identifies and delineates priority by combining multiple criteria, such as species richness, habitat vulnerability, and anthropogenic pressures, etc., for conservation areas. For instance, Regan et al. [33] propose that the MCA approach can integrate ecological, social, and economic data to provide a scientific basis for biodiversity conservation. In a similar vein, Grêt-Regamey et al. [34] have developed spatial decision-support tools to integrate ecosystem services into spatial planning and optimize the layout of protected areas. Thomas Ranius et al. [35] have emphasized the need for protected areas to be selected and managed with consideration of ecological, social, and governance criteria to ensure their effectiveness in the context of changing environmental conditions.

In this study, biodiversity hotspots in the Indo-Pacific convergence zone were identified through a spatial analysis approach which combined several criteria, including ecological sensitivity, biodiversity richness, and anthropogenic pressure. In this study, we examined the effectiveness of protected area networks by integrating multiple biological, ecological, and economic factors. In this study, five distinct criteria were examined to assess the intrinsic value of biodiversity in the designated area. These criteria emphasize the value of important habitats, species diversity, threatened species, and endemic species. The adoption of this approach for the selection of sites of potential interest is advantageous in that it facilitates the transparent evaluation of the criteria, offers alternatives, and permits the incorporation of new data as they become available.

### 4.2. Human Activities and Biodiversity Linkage

In recent years, with the continuous progression of remote sensing technology and geographic information system (GIS) technology, spatial analysis has become an important tool for biodiversity conservation [36]. For instance, the utilization of GIS technology has been demonstrated to facilitate the effective integration of diverse ecological data sources, thereby providing intuitive spatial distribution information that offers substantial support for the planning of protected areas [36]. Furthermore, the widespread adoption of Global Positioning Systems (GPSs) and remotely sensed data has led to the incorporation of spatial analyses in numerous marine ecological conservation studies worldwide [13].

In this study, biodiversity hotspot areas (red areas) and anthropogenic pressure hotspot areas (blue areas) in the Indo-Pacific convergence zone were superimposed by spatial analysis techniques (Figure 5). The results indicate that there is reduced spatial overlap between these two hotspot areas, suggesting that there is a significant spatial separation between biodiversity hotspot areas and anthropogenic pressure hotspot areas in the IPCZ. This finding is consistent with the study by Renema et al. [6], which suggested that the distribution of biodiversity hotspots is somewhat migratory and closely related to geological changes, climate changes, and human activities.

There are several potential explanations for this spatial separation. Firstly, biodiversity hotspots are species occurrence records characterized by the presence of fragile ecosystems, such as coral reefs and mangroves, which exhibit a high degree of ecological vulnerability and consequently possess a heightened susceptibility to threats. For instance, the Raja Ampat archipelago, recognized as the global epicenter of tropical marine biodiversity, is distinguished by its abundant coral and fish species, thus classifying it as one of the most significant coral reef ecosystems on the planet. The geographic isolation and limited accessibility of the area provide a natural barrier to its unique biodiversity and pristine landscape, thereby reducing disturbance from human activities [37]. This phenomenon is also validated in the present study, where some biodiversity hotspot areas are located in relatively closed marine regions which are less affected by shipping and fishing activities.

Secondly, anthropogenic pressure hotspots are usually concentrated in areas of high economic activity, mainly from shipping and fishing activities, such as the Malacca Straits, one of the most important shipping lanes in the world which carries a large amount of international trade. When organisms are compelled to evade these pressures they often migrate to areas where exploitation is less concentrated [37]. A pertinent example is that of the Nassau grouper, which, due to the collapse of its historical spawning aggregations, has been compelled to migrate to new spawning grounds [38]. Research has demonstrated that shipping noise can disrupt fish communication and behavior, particularly in species that rely on sound for navigation and social interactions [39]. Specifically, the acoustic disturbance caused by shipping has been demonstrated to interfere with the vocalizations of fish, which are essential for their survival. This finding is consistent with the results of this study, which indicate that certain biodiversity hotspots are located further away from areas of heavy shipping. This suggests that organisms may be exhibiting avoidance behavior in response to anthropogenic pressures.

In summary, the results of this study indicate that the spatial separation of biodiversity hotspots from areas of anthropogenic stress not only reflects the vulnerability of ecosystems but also reveals the impacts of anthropogenic activities on these areas. This finding is consistent with Halpern et al. [4], who identified overfishing and shipping activities as the two main factors currently affecting biodiversity in the Indo-Pacific convergence zone. Consequently, a comprehensive understanding of the distribution of anthropogenic pressures in the Indo-Pacific region is imperative for the effective implementation of targeted biodiversity conservation strategies.

### 4.3. Biodiversity Priority Conservation Areas and the Impact of Conservation Gaps

The identification of biodiversity priority conservation areas (BPAs) is one of the core elements of marine conservation planning. These areas are characterized by exceptionally high biodiversity values and frequently represent the focal point of conservation initiatives. However, there are still significant gaps in the coverage of these priority areas in the current conservation network. This study found that only 13.88% of the biodiversity hotspots located in existing MPAs had been identified, indicating that most of the biodiversity hotspots had not yet been effectively included in the conservation network.

The economic interests of stakeholders must be considered when planning for marine conservation, especially in areas with high shipping and fishing activities. To achieve a harmonious balance between biodiversity conservation and economic interests, this study proposes the following strategies. First, when identifying priority biodiversity conservation areas, major shipping and fishing routes should be avoided as much as possible, and areas with high conflict with existing economic activities should be avoided. The utilization of spatial analysis techniques facilitates the identification and prioritization of areas exhibiting minimal conflict, thereby ensuring the judicious allocation of conservation resources. Secondly, for critical areas that cannot be avoided completely, such as the island areas of Phuket, Singapore, the Straits of Lombok, Manila in Luzon, Darwin, and Gomogomo, Longar, and others shown in Figure 5B, a balanced strategy is necessary because of the harbors and shipping lanes involved in these areas. Measures such as the establishment of buffer zones, the implementation of temporal or spatial no-take zones, the imposition of speed limits in shipping lanes, and the implementation of shipping controls can be implemented in these areas to mitigate conflicts between protected areas and economic activities. Additionally, the promotion of green shipping technology [40] and the adoption of low-emission ships can effectively mitigate the ecological impact of shipping activities. Concurrently, the implementation of fishing quota systems can contribute to the sustainability of marine ecosystems. Research has been conducted to assess the ecological effects of no-take zones, and it found that fish abundance and density in no-take zones were significantly higher than in non-protected areas [41]. This fully demonstrates the effectiveness of no-take zones in protecting marine biodiversity. Additionally, it is imperative to impose limitations on vessel speeds to ensure the conservation of North Atlantic right whales. Research has demonstrated a direct correlation between the speed of vessels and the probability of fatal collisions with whales [42]. However, the implementation of these measures is encumbered by numerous challenges [43,44], necessitating a careful balancing act between the shipping industry and the imperative for marine ecological protection to ensure the effective implementation of these measures.

This study proposes a methodology for identifying significant regions for biodiversity conservation, thereby providing a scientific framework for marine biodiversity conservation planning in the Indo-Pacific convergence zone. This approach integrates ecological criteria, fisheries shipping data, and spatial analysis techniques to facilitate informed decision-making. In future research and practice, the systematic conservation planning method, in which different weights are set for different input layers to more accurately identify and prioritize conservation areas, should be further explored and applied. This approach promises not only to enhance the scientific and effective dimensions of conservation planning, but also to optimize the coordination of the relationship between biodiversity conservation and human activities. Furthermore, the integration of marine biodiversity informatics in prioritizing conservation is supported by this study through the analysis and integration of extensive biodiversity data, thus providing more comprehensive and precise information to support conservation planning.

## 5. Conclusions

This study demonstrates that the Indo-Pacific Convergence Zone harbors critical yet underprotected biodiversity hotspots, with only 6% of its area currently under formal protection. By synthesizing ecological vulnerability, species richness, and human pressure data, we identified spatial mismatches between biodiversity hotspots and anthropogenic activity centers, driven by habitat sensitivity and economic activity clustering. The proposed priority conservation areas, which focus on regions such as the southern coasts of the Philippines and Sulawesi in Indonesia, aim to address urgent gaps in the existing MPA network. Strategic measures, such as temporal fishing closures and shipping lane speed limits, could mitigate conflicts between conservation and resource use. Future efforts must integrate dynamic governance frameworks and real-time monitoring technologies to adapt to shifting species distributions under climate change. This multi-criteria approach offers a replicable model for transboundary marine spatial planning, advancing global biodiversity targets while supporting sustainable ocean economies.

## Figures and Tables

**Table 1:** Data used in this study.

Data Category	Data Features	Data Source and Date
Fragile and Sensitive Habitat		
Coral reef	Coral reef distribution	UNEP-WCNC (https://resources.unep-wcmc.org/; assessed on 10 June 2024)
Mangrove	Mangrove distribution	UNEP-WCNC (https://resources.unep-wcmc.org/; assessed on 9 June 2024)
Seagrass	Seagrass distribution	UNEP-WCNC (https://resources.unep-wcmc.org/; assessed on 18 June 2024)
Biological Diversity		
Species occurrence	A total of 15,045 species of 5 taxa	OBIS (https://obis.org/; assessed on 9 October 2024)
Species ranges	A total of 7231 species of 5 taxa	AquaMaps (https://www.aquamaps.org/; assessed on 12 October 2019)
Species of Conservation Concern		
Species occurrence	A total of 946 species of 5 taxa, with 91,038 records	IUCN (https://iucn.org/; assessed on 1 April 2024); CITES (https://cites.org/eng; assessed on 25 May 2024)
Restricted-Range Species		
Endemic reef fishes distribution	A total of 373 species	Allen (2008) [9]
Human Ocean Activities		
Apparent fishing effort	2021–2023	https://globalfishingwatch.org/ (accessed on 25 May 2024)
Vessel presence	2021–2023	https://globalfishingwatch.org/ (accessed on 25 May 2024)
Marine Protected Areas (MPA)		
	Coverage of MPAs	https://www.protectedplanet.net/en (accessed on 25 May 2024)

**Table 2:** The proportion of the modeled geographical distribution ranges of five taxonomic groups of marine species.

Taxonomic Groups	Total Number of Known Species in World	Species Analyzed		Occurrence Frequency (%)	Species Per Cells	
		Number	Proportion of Global Total (%)		Maximum	Mean
Actinopterygii	32,513	4763	14.65	90	3824	1064
*Bony fishes*						
Anthozoa	7073	611	8.64	66	607	195
*Corals* and *anemones*						
Elasmobranchii	1226	262	21.37	90	161	58
*Sharks*, *rays*, *sawfish*						
Mammalia	5852	34	0.58	87	28	11
*Whales*, *dophins*, *dugongs*						
Mollusca	57,772	1561	2.70	90	2063	456
*Gastropods*, *cephalopods and bivalves*						
Total	104,436	7231	6.92			

Based on Catalogue of Life (https://www.catalogueoflife.org/annual-checklist, 8 November 2024).

**Table 3:** The number of species of conservation concern, as determined by the International Union for Conservation of Nature (IUCN) Red List and the Convention on International Trade in Endangered Species of Wild Fauna and Flora (CITES).

Category	Number of Species					Total
	Actinopterygii	Anthozoa	Elasmobranchii	Mammalia	Mollusca	
IUCN Red List						
CR-Critically Endangered	1	3	25	0	0	29
EN-Endangered	16	10	18	4	0	48
VU-Vulnerable	45	158	21	7	6	237
NT-Near Threatened	28	145	7	3	3	186
LC-Least Concern	4	162	7	19	1	193
DD-Data Difficent	5	20	0	2	2	29
CITES						
I-Appendix I	1	0	6	17	0	24
II-Appendix II	20	710	76	19	13	838
Total ^a^	99	710	82	36	19	

^a^ Some species are listed in several categories.

## Data Availability

This research did not generate any new data. The dataset supporting the findings of this research is available upon request.

## Supplementary Materials

The following supporting information can be downloaded at: https://www.mdpi.com/article/10.3390/biology14060700/s1, Figure S1: Vessel pressures and apparent fishing effort; Table S1: Species occurrence records; Table S2: Species range records; Table S3: Protected species.
