# Supplementary figures and images for: Marine Biodiversity Conservation Planning in the Indo-Pacific Convergence Zone Based on Ecological Spatial Analysis
Source: Biology (Basel). 2025 Jun 14;14(6):700. doi: 10.3390/biology14060700 (PMC12189379; doi:10.3390/biology14060700)

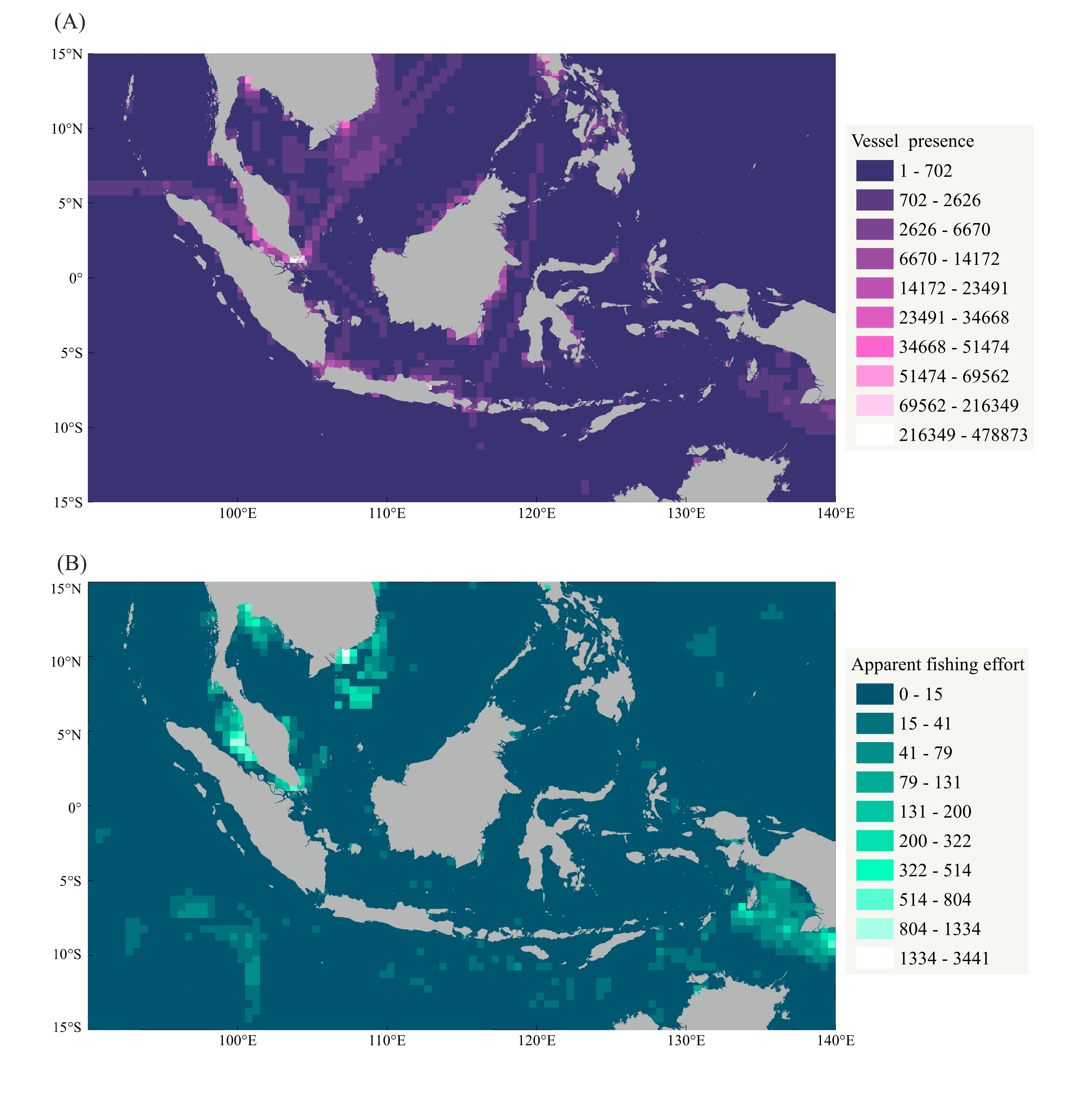

Supplement: Supplementary file 1 [file biology-14-00700-s001.zip › FigureS1 Vessel Pressures and Apparent Fishing effort.tif]
